# Silver nanoparticles biosynthesis using mixture of *Lactobacillus* sp. and *Bacillus* sp. growth and their antibacterial activity

**DOI:** 10.1038/s41598-024-59936-1

**Published:** 2024-05-03

**Authors:** Morad G. S. S. Al-asbahi, Bashir A. Al-Ofiry, Fuad A. A. Saad, Adnan Alnehia, Murad Q. A. Al-Gunaid

**Affiliations:** 1 Department of Biology, Faculty of Sciences, Sana’a University, 12081, Sana’a, Yemen; 2 Department of Biology, Faculty of Applied Sciences, Thamar University, 87246, Dhamar, Yemen; 3 Department of Physics, Faculty of Applied Sciences, Thamar University, 87246, Dhamar, Yemen; 4 Department of Chemistry, Faculty of Education, Thamar University, 87246, Dhamar, Yemen

**Keywords:** Chemical biology, Microbiology

## Abstract

The biosynthesis of nanoparticles offers numerous advantages, including ease of production, cost-effectiveness, and environmental friendliness. In our research, we focused on the bioformation of silver nanoparticles (AgNPs) using a combination of *Lactobacillus* sp. and *Bacillus* sp. growth. These AgNPs were then evaluated for their biological activities against multidrug-resistant bacteria. Our study involved the isolation of *Bacillus* sp. from soil samples and *Lactobacillus* sp. from raw milk in Dhamar Governorate, Yemen. The synthesized AgNPs were characterized using various techniques such as UV–visible spectroscopy, X-ray diffraction (XRD), Fourier-transform infrared spectroscopy (FTIR), and transmission electron microscopy (TEM). The antibacterial properties of the AgNPs were assessed using the modified Kirby Bauer disk diffusion method against multidrug-resistant strains of *Staphylococcus aureus* and *Pseudomonas aeruginosa*. Our results demonstrated that the use of a bacterial mixture for biosynthesis led to faster and more effective production of AgNPs compared to using a single bacterium. The UV–visible spectra showed characteristic peaks indicative of silver nanoparticles, while XRD analysis confirmed the crystalline nature of the synthesized particles. FTIR results suggested the presence of capping proteins that contribute to the synthesis and stability of AgNPs. Furthermore, TEM images revealed the size and morphology of the AgNPs, which exhibited spherical shapes with sizes ranging from 4.65 to 22.8 nm. Notably, the antibacterial activity of the AgNPs was found to be more pronounced against *Staphylococcus aureus* than *Pseudomonas aeruginosa*, indicating the potential of these nanoparticles as effective antimicrobial agents. Overall, our study highlights the promising antibacterial properties of AgNPs synthesized by a mixture of *Lactobacillus* sp. and *Bacillus* sp. growth. Further research is warranted to explore the potential of utilizing different bacterial combinations for enhanced nanoparticle synthesis.

## Introduction

The topic of nanoparticles in particular has attracted attention in a variety of areas in recent years. The word “nano” refers to a dimension in the order of a billion (10^9^) metres and is derived from the Greek term “nanos”, which means dwarf. The surface-to-volume ratio is the most important property of nanoparticles, enabling them to interact more quickly with other particles^1^.

Due to the high biological, physical and chemical properties of silver nanoparticles, which are widely researched and used in medicine, cultivation and environmental remediation, the synthesis method for silver nanoparticles has recently undergone significant development^2–5^. These properties of silver nanoparticles are mainly due to the crystallinity, composition, size, shape and structure of silver nanoparticles compared to their large form^6^. Silver nanoparticles (AgNPs) have high antibacterial activity against susceptible and multi-drug resistant (MDR) bacterial strains^7^, so they have long been used as broad-spectrum antimicrobials against many pathogenic and non-pathogenic microbes in various industrial areas such as textiles and food packaging^8^. The use of antibiotics is becoming increasingly ineffective due to the development of multi-resistant strains of bacteria^9^, which means that innovative techniques are needed to cure bacterial infections. Silver nanoparticles are substances used in these techniques^10^.

There are various methods used to synthesis silver nanoparticles, including biological, chemical and physical methods. However, the chemicals used in many of these methods are toxic, flammable and not easily removed, posing a risk to individuals and the environment^11,12^. Biosynthesis seems to be a promising area to synthesize nanoparticles in an environmentally friendly, simple, safe, cost-effective and time-saving way^13–15^. Fungi, bacteria, algae and plants are increasingly being used to biosynthesize nanoparticles^16^. Interestingly, when silver nanoparticles are synthesized from silver metal using biological methods, it has been observed that their antimicrobial activity increases while their toxicity decreases^17^. Some of the bacteria that have been used on a large scale for the bioformation silver nanoparticles are *Escherichia coli*, *Lactobacillus casei*, *Aeromonas* sp. *Pseudomonas proteolytica*, *Bacillus cereus*, *Bacillus* subtilis, *Bacillus amyloliquefaciens*, *Bacillus indicus*, and *Bacillus cecembensis*^18^. Bacteria synthesize nanoparticles in different ways, depending on the type of bacteria. Some bacteria synthesize nanoparticles via extracellular synthesis and other bacteria via intracellular synthesis^1^.

The reduction of metal ions to NPs by plant extractors is swifter than the use of microorganisms^19^, but the synthesis of nanoparticles by plant extractors may require heating to 85 °C, which can be expensive when large amounts of nanoparticles are synthesized^1^. Not getting raw materials and harvesting the plants at the wrong time may be also a challenge to biosynthesis of the nanoparticles by plant extractors^20^. To improve the efficiency of microorganism in biosynthesis, pharmaceuticals production, biodegradation and food industry, a mixture of microorganisms is used, which is called coculture, co-culture, binary culture, heteroculture, heterotypic culture, mixed culture, dual culture, bi-culture, tri-cultivation, co-localization or co-cultivation. The System of mixed cultivation will be fundamental importance for the advancement of synthetic biology. The study of natural interaction between cells will spotlight new ways of reengineering even in organisms that are difficult to cultivate^21^. The synthetic/artificial co-culture systems surpass the limitations of monocultures or consortia in nature with the added advantages in exploring allelopathic interactions in food industries involving fermentation and natural product/drug discovery^22^. The use of a mixture of different microorganism cultures is gaining great interest in bioleaching, decontamination and bioelectrical production, where the use of a single microorganism has so far not yielded good results^23^. The results of Grujić et al.^24^ show that the mixed biofilm (*Escherichia coli* and *Rhodotorula mucilaginosa*) has the highest efficiency in the removal of Zn2^+^ (99.26%), Pb2^+^ (99.52%) and Cu2^+^ (99.88%). The metal removal efficiency was within the limits of 94.99–99.88% for the mixed biofilm and 81.56–97.85% for the single biofilm. The experiments of Yang et al.^25^ have shown that the degradation of antibiotics in wastewater is better than the use of a single antibiotic and that the use of mixed aerobic and anaerobic conditions practerial species. Nwokoro and Dibua^26^ also found that the use of a mixture of *Pseudomonas stutzeri* and *Bacillus subtilis* was more effective in removing cyanide from soil than the use of a single bacterium.

Most studies on the biosynthesis of silver nanoparticles by bacteria using a bacterial genus. Dakhil^27^ used a mixture of *Lactobacillus* species and found that silver nanoparticles were biosynthesized by a mixture of *Lactobacillus acidophillus* and *Lactobacillus plantarum* and had a typical shape (spherical) and a size of 30–100 nm, he also found that the distribution of silver nanoparticles formed by this mixture was more consistent than the nanoparticles formed by a mixture of *Lactobacillus acidophillus* and *Lactobacillus bifidus*.

The use of more than one bacterial genus in the synthesis of silver nanoparticles can improve and accelerate the synthesis process. Therefore, in the present study, the synthesis of AgNPs by mixing the growth of *Lactobacillus* sp. and *Bacillus* sp. was investigated and the biological activities of AgNPs against multidrug-resistant bacteria were evaluated.

## Materials and methods

### Materials

AgNO_3_, nutrient agar, nutrient broth, MRS agar, Mueller–Hinton agar, UIM medium, methyl red (MR) medium, egg yolk agar and Simmon citrate medium from Himedia Laboratories. Carbohydrate discs, H_2_O_2_, Gramme stain and Ziehl-Nelseen stain. Sterile physiological solution (NS; 0.9 w/v% sodium chloride; pH 4.5–7.0). Tubes, absorbent cotton, swabs and loops. Deionized water (DW) was used to prepare the culture media and wash the silver nanoparticles.

### Isolation and identification of bacteria

The soil samples were collected in the vicinity of the car repair shops in Dhamar City, Yemen. The collected soil samples were transported to the laboratory in sterile polyethylene bags. Ten grams of soil were soaked in 90 ml of sterile physiological solution (0.9% w/v) and to kill non-spore forming bacteria, the soil solution was treated in a water bath at 60 °C for one hour to isolate *Bacillus* sp. fill loop from each of the soil solutions, was cultured by swabbing on nutrient agar medium and incubated at 35 °C for 1 day^28^. The raw milk samples from the cows were collected in rural Dhamar Governorate and stored in a sterilized glass bottle. The milk samples were stored at 4 °C and then transported to the laboratory. After the formation of whey in the milk, *Lactobacillus* sp. was isolated by plating the whey milk on MRS agar whose PH was modulated to 5.4 by acetic acid to facilitate the isolation of the genus *Lactobacillus* sp.^29^. The isolated bacteria were identified on the basis of their colonies, their form on the culture media, Gram staining, biochemical and physiological characteristics according to the procedures described in Bergey's Manual of Systematic Bacteriology^30,31^.

### Bacteria growth in nutrient broth

*Lactobacillus* sp. was cultivated in a flask with 100 ml nutrient broth and a flask with 50 ml nutrient broth. The same was repeated with *Bacillus* sp. All flasks were incubated at 30 °C on a shaker and shaken at 120 rpm for 24 h.

### Bio-synthesized silver nanoparticles

After the bacteria had passed into the logarithmic phase, silver nitrate was added to the flask containing 100 ml of *Lactobacillus* sp., to the flask containing 100 ml of *Bacillus* sp. and to the flask containing 100 ml of mixed growth of *Lactobacillus* sp. and *Bacillus* sp., the concentration of silver nitrate in each flask being 2 mM. The flasks were incubated at 30 °C on a shaker at 140 rpm for 24 h to obtain the reaction, noting a color change in the flasks every half hour. This work was carried out twice. The color change of the solution determines the formation of AgNPs. To obtain silver nanoparticles from the growth medium solution, the solution was centrifuged at 10,000 rpm for 20 min. To prevent any component of the growth medium from influencing the properties of the nanoparticles, they were washed three times with distilled water. The silver nanoparticles were dried at 70 °C for 15 h and processed into fine powder for their characterization^32,33^.

### Characterization

The silver nanoparticles were analyzed using different analytical techniques, i.e. UV–visible, X-ray diffraction (XRD), Fourier transform infrared (FTIR) and transmission electron microscopy (TEM). Five ml (5 mL) of the supernatant of the reaction solution was analyzed using a UV–Vis spectrophotometer (Hitachi, Tokyo, Japan) at room temperature to determine the optical properties of the silver nanoparticles. The powder samples of silver nanoparticles were subjected to X-ray diffraction (XRD) analysis. The XRD style was registered by an XD–2 X-ray diffractometer (Beijing Purkinje General Instrument Co., Ltd., Beijing, China) with CuKα radiation of λ = 1.5418Å, over 2-theta 15–75° and scanning rate of 0.02 min^−1^. Fourier-Transform Infrared was used to identify the interaction between biomolecules and AgNPs. FTIR pattern was registered on FTIR in the extent of 400–4000 cm^−1^ at a precision of 4 cm^−1^. AgNPs shapes were evaluated by TEM. Diluted sliver nanoparticles were placed onto carbon-coated copper TEM meshs.

### Antibacterial activity

The antibacterial activities of AgNPs were investigated using the modified Kirby-Bauer disk diffusion method^34^. The antibacterial test was carried out against multi-resistant *Staphylococcus aureus* and *Pseudomonas aeruginosa*. After activation of the bacteria by cultivation on nutrient agar for 24 h and separate turbidity of the bacteria of each species in sterile normal saline solution, the turbidity corresponded to the turbidity of barium sulfate (turbidity standard according to McFarland 0.5). Muller–Hinton agar plates were inoculated with a sterile swab with bacterial turbidity. Sterilized filter leaf disks with a diameter of 5 mm impregnated with biosynthetic silver nanoparticles of different concentrations (10, 20 and 40 μg) were placed on the Muller–Hinton agar plates. The inhibition area was measured in mm to determine the inhibition zone.

### Hemolytic assay

In order to evaluate the biological safety of silver nanoparticles biosynthesized by bacteria, these particles were added to red blood cells in different concentrations, according to Guowei et al.^35^ with a slight modification of his method. Half a millilitre of fresh blood was taken from a young person of group O^+^ in a tube containing an anticoagulant. Then the red blood cells (RBCs) were separated by centrifugation at 5000 rpm for 10 min, the plasma was discarded and the blood cells were washed three times with normal saline. The erythrocyte suspension was then prepared in physiological solution (pH 4.5–7.0) to obtain a 2% cell suspension. Silver nanoparticles were prepared in physiological solution to final concentrations of 3.9–500 μg/ml. 0.5 ml of the erythrocyte suspension was added to 9 tubes, then 0.5 ml of different concentrations of silver nanoparticles were added separately to 7 tubes, while 0.5 ml of the physiological solution was added to tube No. 8 as a negative control and 0.5 ml of distilled water as a positive control. After incubation for 1 h at 37 °C and gentle shaking, the tubes were placed in the centrifuge for 5 min at 5000 rpm and then the hemolysis was measured with the spectrometer at 540 nm.

The hemolytic activity was evaluated by the following equation:

$$ {\text{Percentage}}\;{\text{of}}\;{\text{hemolysis}} = \left( {{\text{A}}_{{\text{S}}} - {\text{A}}_{{\text{N}}} /{\text{ A}}_{{\text{P}}} - {\text{A}}_{{\text{N}}} } \right) \times {1}00 $$

where A_S_ is the absorbance of the test sample, A_N_ is the absorbance of the negative control and A_P_ is the absorbance of the positive control.

## Results and discussion

### Isolated bacteria features

Morphological characterization revealed that both the cultures of *Lactobacillus* sp. and *Bacillus* sp. contained Gram-positive bacilli. Biochemical characterization revealed that both the *Lactobacillus* sp. and *Bacillus* sp. (Table 1).

**Table 1:** Biochemical characterization of *Lactobacillus* sp. and *Bacillus* sp.

Test	*Lactobacillus* sp.	*Bacillus* sp.
Shape	Rod	Rod
Gram stain	+	+
Motility	−	+
Catalase test	−	+
Sporing	−	+
Anaerobic growth		−
Voges proskauer	−	+
Citrate Utilization		+
Egg yolk reaction		−
Fermentation		
Arabinose	+	+
Lactose	+	
Mannose	+	
Mannitol	−	+
Sorbitol	+	
Fructose	−	
Galactose	−	
Xylose		+

+  = positive; − = negative.

### Silver nanoparticles biosynthesis

**Figure 1:**
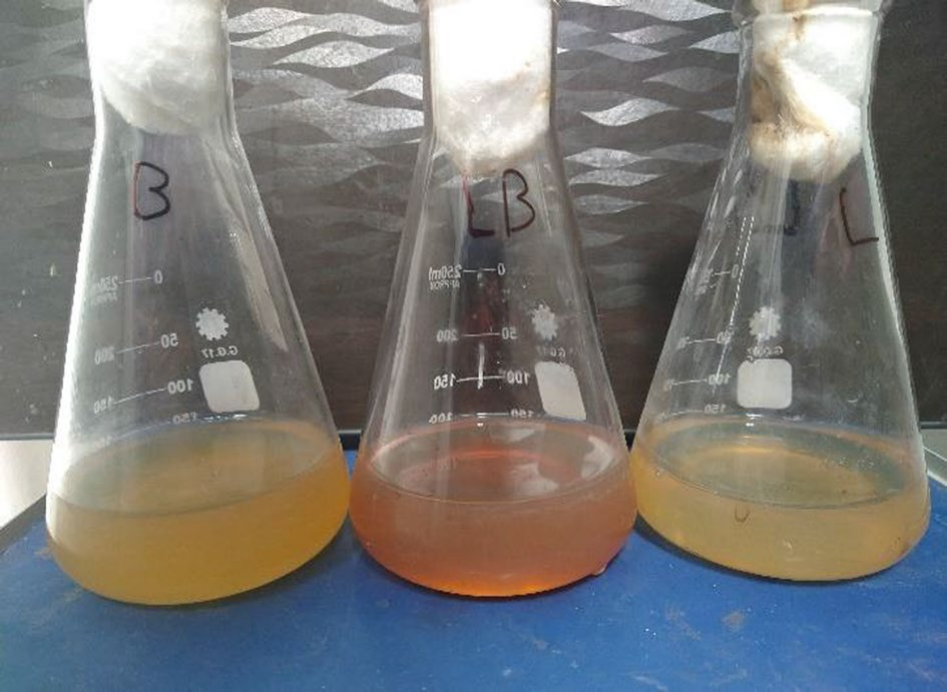
Color change to a dusky orange in the middle flask that contains a mixture of *Lactobacillus* sp. and *Bacillus* sp. growth which indicates the formation of AgNPs.

The ability to biosynthesis of silver nanoparticles by the culture of *Lactobacillus* sp. and *Bacillus* sp. separately was showed, and the ability to biosynthesis of silver nanoparticles by mixture of *Lactobacillus* sp. and *Bacillus* sp. growth also was showed. The silver nanoparticles formation in the culture liquid is observed by the appearance of brown color^36^. The brown color appeared in the aqueous solution of AgNPs due to agitation by surface plasmons^37^. In the flask containing a mixture of *Lactobacillus* sp. and *Bacillus* sp., a dark orange color appeared within less than 1 h (Fig. 1), while in the flasks containing only *Lactobacillus* sp. and *Bacillus* sp., the appearance of a dark orange color was observed only after 16 h. The use of a mixture of bacteria for biosynthesis is faster and more effective than the use of a single bacterium. This could be due to the fact that the metabolic products of one bacterium can be utilized by other bacteria and that the interaction between the bacteria increases the effectiveness of biosynthesis^23,38^. In a study on the biosynthesis of AgNPs by *Bacillus cereus*, the color changed to deep orange after the addition of AgNO_3_ to the *Bacillus cereus* supernatant after 24 h^39^. In another study, the Ag ions (Ag^+^) were minimized to AgNPs within 18 h after the addition of AgNO_3_ to the cell-free supernatant of *Bacillus subtilis*^40^.

### Characterization of AgNPs

#### UV–VIS analysis

**Figure 2:**
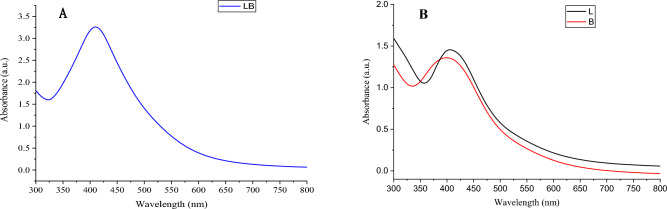
UV–Vis absorption spectra of AgNPs synthesized using (**A**) mixture *Lactobacillus* sp. and *Bacillus* sp. growth (**B**) the growth each bacteria individually.

The bioformation of silver nanoparticles was characterized by UV–visible spectroscopy in the range of 200–500 nm^41^. In our study, we observed a broad peak in the range between 410 and 430 nm in silver nanoparticles biosynthesized by bacteria due to their surface plasmon resonance absorption band (Fig. 2). Our results are close to the findings of previous studies, which suggested that a common AgNps surface plasmon resonance (SPR) absorption band exists at a wavelength in the range of 400–480 nm^42^. Our study showed that the peak of silver nanoparticles formed by mixed growth of *Lactobacillus* sp. and *Bacillus* sp. was sharper and more intense (absorbance at 3.2) (Fig. 2A). Increasing the number of nanoparticles synthesized in aqueous solution leads to an increase in the intensity of the SPR peak^27^.

##### Surface plasmon resonance and UV–Vis spectra analysis according to time

**Figure 3:**
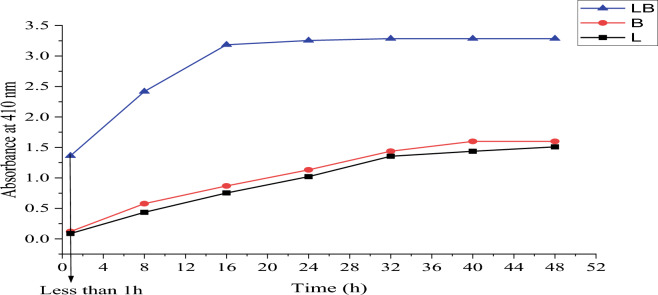
Rate synthesis silver nanoparticles according to time.

After the appearance of a dusky orange color in the flask containing a mixture of *Lactobacillus* sp. and *Bacillus* sp. 5 ml of each flask was sampled and measured by UV–visible spectroscopy and measured every 8 h Fig. 3.

When measured by UV at less than an hour of incubation, we found that silver nanoparticles were not formed by each bacterium separately, while we found the formation of nanoparticles when using a mixture of *Lactobacillus* sp. and *Bacillus* sp. growth. The maximum time to complete the synthesis of silver nanoparticles by a mixture of *Lactobacillus* sp. and *Bacillus* sp. growth was 16 h. The maximum time for complete synthesis by *Bacillus* sp. alone is 40 h and more than 40 h by *Lactobacillus* sp. alone. The passage of reaction time increases the intensity of absorption until the reaction is completed^43^. Lv et al.^44^ found that the depletion of silicon from electrolytic manganese residues with a mixture of *Paenibacillus mucilaginosus* and *Bacillus circulans* was better and faster than using the individual bacteria.

In their study, El-Saadony et al.^45^ found that the maximum time for the synthesis of silver nanoparticles by *Bacillus subtilis* ssp *spizizenii* MT5 was 40 h. In another study, Tariq et al.^46^ found that the reaction in the synthesis of silver nanoparticles by *Bacillus subtilis* was completed after 72 h. Matei et al.^47^ reported in their study that the synthesis of AgNPs by *Lactobacillus plantarum* was completed after 24 h, while Chaudhari et al.^48^ found that the reaction was completed after 3 days when 2 isolates of *Lactobacillus* species were used.

#### XRD of AgNPS

**Figure 4:**
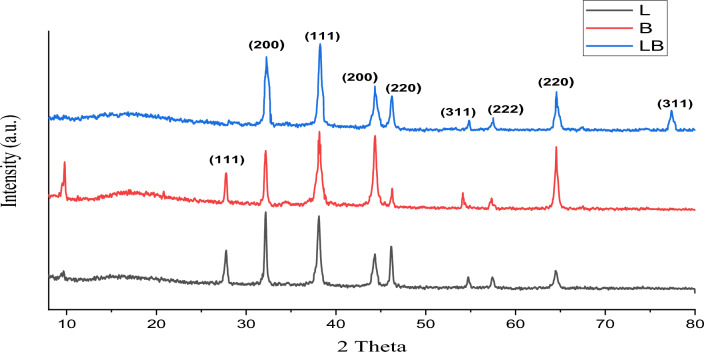
XRD spectra of AgNPs synthesized using mixture of *Lactobacillus* sp. and *Bacillus* sp. growth (LB) and *Lactobacillus* sp. and *Bacillus* sp. growth separately (L and B).

The crystalline properties and purity of the silver nanoparticles synthesized by bacteria were determined by XRD. The X-ray diffraction pattern of silver nanoparticles biosynthesized by the growth of *Lactobacillus* sp. and *Bacillus* sp. Nine peaks were detected on XRD at 2θ values of 27.87°, 32.32°, 38.23°, 44.44°, 46.22°, 54.8°, 57.34°, 64.63° and 77.39°, which correlate with the reflectance of (111), (200), (220), (222) and (311), respectively (JCPDS-ICDD files No 04-0783), as shown in Fig. 4. Our results were close to the results of previous studies on the characterization of AgNPs by XRD^17,49^. The purity of silver nanoparticles was achieved in the absence of other external peaks, suggesting that the use of two types of bacteria is an effective method for obtaining high-purity silver nanoparticles. However, the undefined peaks at 2θ° of 9.538 and 10.45 in the L and B profiles, respectively, could be due to some organic matter in the growth medium^50^. Regarding the silver nanoparticles biosynthesized in our study, we found that the particles biosynthesized by a mixture of *Lactobacillus* sp. and *Bacillus* sp. bacteria were purer than the particles synthesized by each bacterium individually, with peaks at 2θ° with values of 38.23°, 44.44°, 64.63° and 77.39° indicating the high purity of silver nanoparticles^17^.

#### FTIR analysis

**Figure 5:**
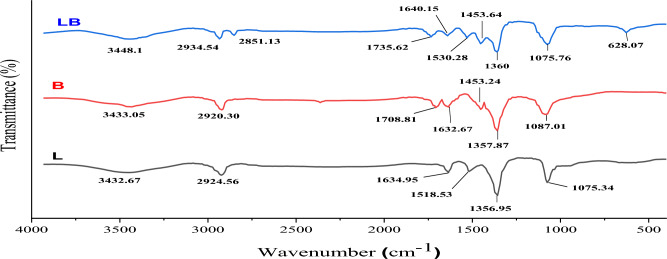
FTIR spectra of AgNPs synthesized using mixture of *Lactobacillus* sp. and *Bacillus* sp. growth (LB) and *Lactobacillus* sp. and *Bacillus* sp. growth separately (L and B).

The dried silver nanoparticles were analyzed by FTIR to determine the presence of a protein around the silver nanoparticles that could be responsible for the synthesis and stability of the silver nanoparticles. Figure 5 shows the FTIR spectra of the nanoparticles synthesized using a mixture of *Lactobacillus* sp. and *Bacillus* sp. (LB), *Lactobacillus* sp. (L) and *Bacillus* sp. (B). The FTIR spectrum of AgNps shows that the peaks at 3448.1, 3433.05 and 3432.67 cm^−1^ are due to the existence of the –NH or –OH group and some free amides^51,52^, but these peaks were weak, indicating the reduction of Ag^+^ by –OH groups^53^ and the amine –NH chelated by Ag^+^ ions^54^.

The observed peaks show from 2935 to 2920 cm^−1^ and 2851.13 cm^−1^ due to the tremolization of the symmetric and asymmetric stretching of the C-H functional group, which represent the status of the aliphatic and aromatic styles^55,56^.

The absorption around1735.62 and 1708.81 cm^−1^ is due to the C = O stretching vibration absorption of amides^57^, and the peaks at 1640.15, 1634.95, and 1632.67 cm^−1^ show the presence of alkenyl C=C in the proteins (amide I)^56^. These peaks appear weak in Fig. 5, indicating the oxidation of C=O and C=C bonds^54^. The absorbance around 1530.28 and 1518.53 cm^−1^ as a result of NH stretching in amide II^53^, and the peaks at 1453.64 and 1453.24 cm^−1^ were attributed to C-H binding of methylene in proteins^53,55^. The strong peaks at 1360, 1357.87 and 1356.95 cm^−1^ are due to aromatic amine or CN stretches. The observed peak at 1075.76 cm^−1^ probably indicates the tensile tremolo of the C–O stretch^58^. FTIR results have demonstrated the presence of a capping protein in silver nanoparticles from growth bacteria^59^. The carboxyl group C=O, the hydroxyl group –OH and the free amine –NH of the bacterial protein may be responsible for the synthesis and stability of the silver nanoparticles^60–62^. Figure 5 shows many convenient groups on the outside of silver nanoparticles synthesized using a mixture of *Lactobacillus* sp. and *Bacillus* sp. compared to silver nanoparticles synthesized by the individual bacteria. In the mixed growth of two types of bacteria, the total amount of protein produced is higher than the protein produced when each bacterium grows separately^63^.

#### TEM

**Figure 6:**
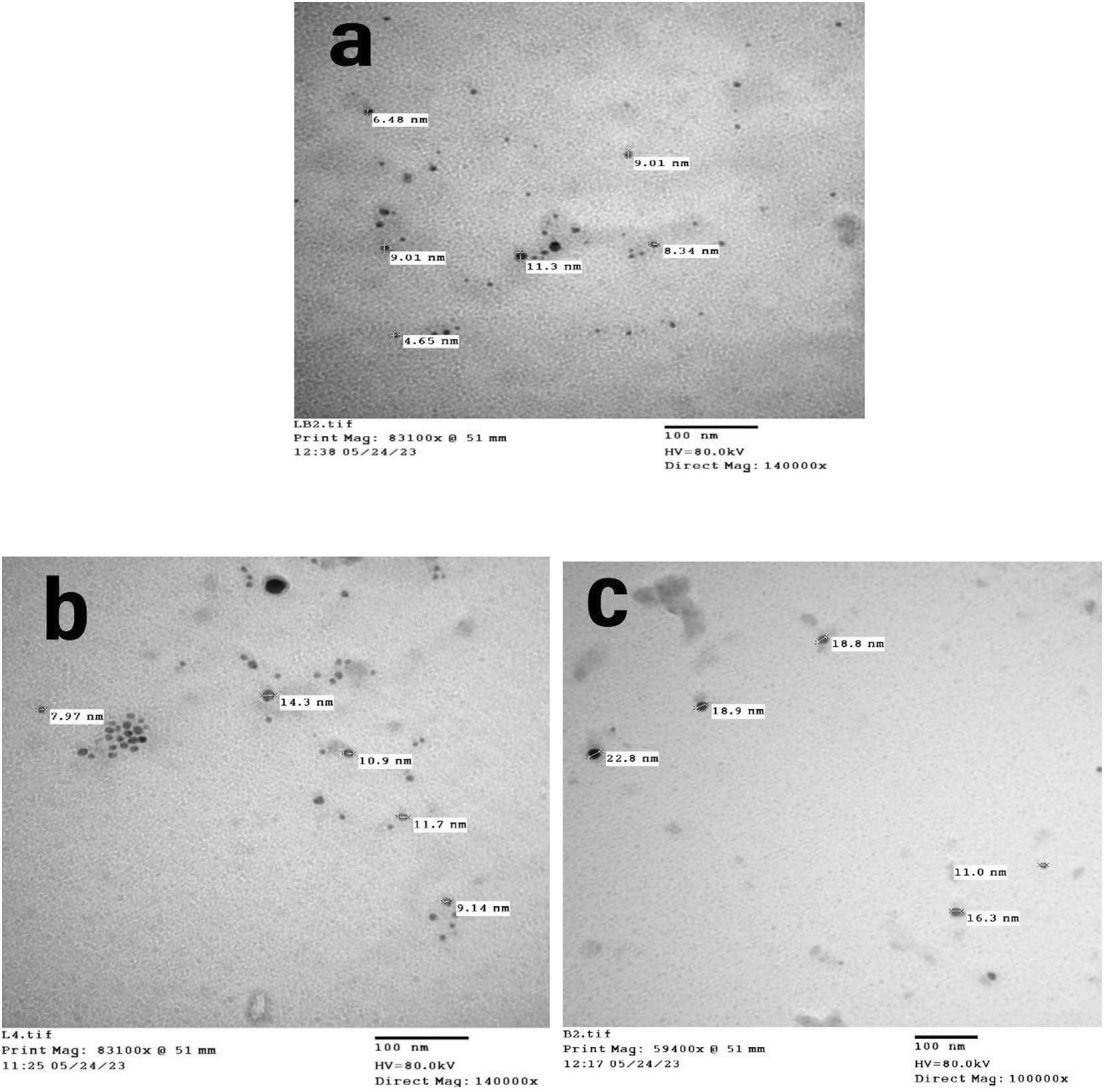
TEM images of AgNPs synthesized by (**a**) mixture *Lactobacillus* sp. and *Bacillus* sp. growth; (**b**) *Lactobacillus* sp. (**c**) *Bacillus* sp.

The TEM image of the silver nanoparticles synthesized by bacteria is shown in Fig. 6. The TEM micrograph shows the size of the silver nanoparticles ranging from 4.65 to 11.3 nm for the nanoparticles synthesized by a mixture of *Lactobacillus* sp. and *Bacillus* sp., 7.97–14.3 nm for the nanoparticles synthesized by *Lactobacillus* sp. and 11–22.8 nm of the nanoparticles synthesized by *Bacillus* sp. Electron micrographs showed that the silver nanoparticles synthesized by a mixture of *Lactobacillus* sp. and *Bacillus* sp. were well distributed, indicating that these nanoparticles are more stable than those synthesized by each bacterium individually. The discrete distribution of AgNPs seen in the TEM could be due to clogging by proteins^36^.

### Inhibition zones of AgNPs on two multidrug resistant bacteria

**Figure 7:**
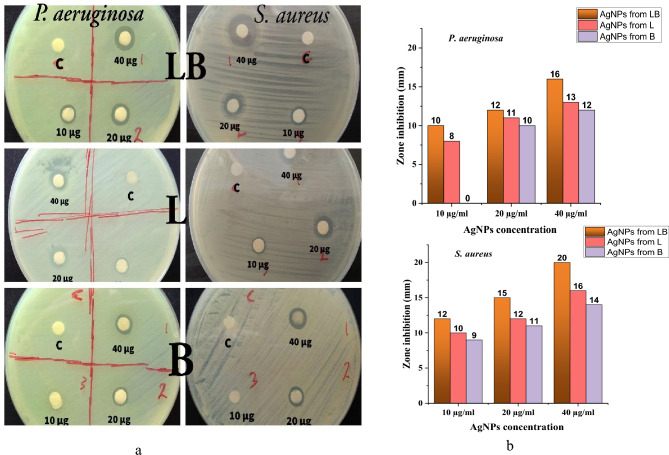
(**a**) Effectiveness tests against bacteria for AgNPs synthesized by a mixture *Lactobacillus* sp. and *Bacillus* sp. (LB), *Lactobacillus* sp. (L) and *Bacillus* sp. (B): (**b**) Diagram showing silver nanoparticles synthesized by a mixture of *Lactobacillus* sp. and *Bacillus* sp. growth had greater antibacterial activity than silver nanoparticles synthesized by each bacteria separately.

The biosynthesized AgNPs were tested for their antibacterial activity against multidrug-resistant bacteria (*Staphylococcus aureus* (Gram positive) and *Pseudomonas aeruginosa* (Gram negative)) isolated from clinical samples. The antibacterial activity was determined using the disc diffusion method at three different concentrations of 10, 20 and 40 μg. The diameter of the inhibition zone (mm) around each disc with silver nanoparticles is shown in Table 2. The silver nanoparticles synthesized with a mixture of *Lactobacillus* sp. and *Bacillus* sp. and each bacterium individually showed antibacterial activity against *Staphylococcus aureus* and *Pseudomonas aeruginosa* (Table 2 and Fig. 7). Our results were very close to the results of previous studies^43,64^.

**Table 2:** Antibacterial activity of AgNPs using the disc diffusion method.

Bacteria		Inhibition zone (mm) (mean ± SD of 3 replicates)			Control negative Ag NO_3_ 40 µg/ml
		10 µg/ml	20 µg/ml	40 µg/ml	
AgNps from LB	*Staphylococcus aureus*	12 ± 0.4	15 ± 0.1	20 ± 0.2	–
	*Pseudomonas aeruginosa*	10	12 ± 0.1	16 ± 0.22	–
AgNPS from L	*Staphylococcus aureus*	10 ± 0.43	12 ± 0.24	16 ± 0.34	–
	*Pseudomonas aeruginosa*	8 ± 0.76	11 ± 0.5	13 ± 0.3	–
AgNPs from B	*Staphylococcus aureus*	9 ± 0.43	11 ± 0.12	14 ± 0.22	–
	*Pseudomonas aeruginosa*	-	10 ± 0.23	12 ± 0.25	–

*LB* *Lactobacillus* sp. and *Bacillus* sp., *L* *Lactobacillus* sp., *B* *Bacillus* sp.

In this study, we found that silver nanoparticles synthesized by a mixture of *Lactobacillus* sp. and *Bacillus* sp. exhibited greater antibacterial activity than silver nanoparticles synthesized by each bacterium individually. This greater efficacy could be due to the small size of the silver nanoparticles synthesized by the bacterial mixture (4.65–11.3 nm). The increase in the effectiveness of silver nanoparticles in killing bacteria depends on the size of the NPs, as smaller NPs have a larger outer surface area, so their effectiveness in killing bacteria is greater than larger NPs^42,63,65,66^, and this effectiveness also depends on the reducing agents and stabilizers^67^.

In our study, the antibacterial activity of AgNPs synthesized by bacteria was higher against *Staphylococcus aureus* than against *Pseudomonas aeruginosa*. The reason that *Pseudomonas aeruginosa* was less susceptible to silver nanoparticles may be that these Gram-negative bacteria have an outer membrane that is difficult to penetrate and accumulate silver nanoparticles in the cell by forming efflux pumps with outer membrane proteins. These efflux pumps excrete harmful substances, including biocides and antibiotics^68^. The mechanism of resistance through efflux pumps may be naturally present in bacteria or may have arisen through mutation or gene transfer^69^.

The increased efficacy of AgNPs against *Staphylococcus aureus* could also be due to the fact that these AgNPs were synthesized by Gram-positive bacteria, as Singh and Mijakovic^8^ found in their study that silver nanoparticles produced by a bacterial species are more effective against bacteria closely related to that species. The effectiveness of silver nanoparticles against microbes is due to several mechanisms, adhesion to the cell wall and plasma membrane of the microbial cell, resulting in increased cell permeability^57^ or destruction of the plasma membrane and leakage of cell components^70,71^. Formation of reactive oxygen species (ROS) and free radicals and modification of microbial signal transduction pathways, which damage DNA and the cell wall and increase the permeability of the plasma membrane^6,72^. Prevention of DNA replication through the release of silver ions^73^ which are bound to the phosphorus in the DNA^74^.

### Hemolytic of AgNPs

**Figure 8:**
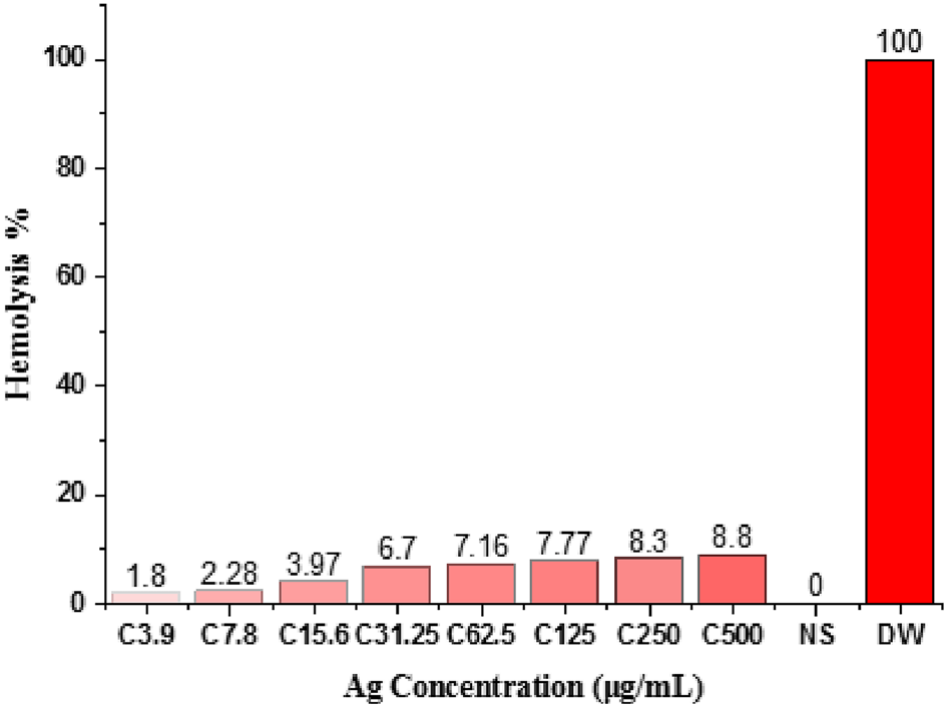
Hemolysis of RBCs caused by AgNPs at different concentrations (3.9–500 μg/mL), physiological solution (negative control), and distilled water (positive control).

The hemolytic assay was performed with different concentrations of AgNPs (3.9–500 μg/ml) to evaluate the potential toxicity of the nanoparticles on human RBCs and used normal saline as negative control and distilled water as positive control. Figure 8 illustrates the averaged data obtained from the two experiments. In our study, the hemolysis of RBCs by AgNPs at concentration 500 µg/ml, 250 µg/ml, 125 µg/ml. 62.5 µg/ml and 31.25 µg/ml were 8.8, 8.3, 7.77, 7.16 and 6.7% respectively, while slightly hemolysis at concentration 15.62 and 7.8 were 3.97 and 2.28% (Table 3). The non-hemolysis effect at 3.9 µg/ml, where the hemolysis was 1.8%. Substances causing hemolysis > 5% were classified as toxic, while those causing hemolysis 2–5% were less toxic and while that cause hemolysis < 2% were non-toxic^50,75^. Our results showed that the silver nanoparticles had no toxicity compared to the silver nanoparticles in the study by Rajora et al.^59^ where they found that hemolysis was 3.58% at a concentration of 0.5 for silver nanoparticles, 4.7% at 1 µg and 8.2% at 2 µg. The rate of hemolysis increases with increasing concentration and small size of the silver nanoparticles^76^.

**Table 3:** Hemolytic efficacy of silver nanoparticles on RBCs.

No	Transactional	Hemolysis
1	500 µg/ml	8.8%
2	250 µg/ml	8.3%
3	125 µg/ml	7.77%
4	62.5 µg/ml	7.16%
5	31.25 µg/ml	6.7%
6	15.6 µg/ml	3.97%
7	7.8 µg/ml	2.28%
8	3.9 µg/ml	1.8%
9	CN (NS)	0
10	CP (DW)	100%

*CN* control negative, *CP* control positive, *NS* normal slain, *DW* distilled water.

## Conclusions

In our study, we observed that the combination of *Lactobacillus* sp. and *Bacillus* sp. in the biosynthesis of silver nanoparticles (AgNPs) resulted in a faster and more effective process than the use of a single bacterium. Interestingly, the AgNPs produced by the mixed growth of *Lactobacillus* sp. and *Bacillus* sp. were found to be purer than those synthesized by each individual bacterium. Furthermore, these AgNPs exhibited enhanced antibacterial properties, particularly against *Staphylococcus aureus*, compared to *Pseudomonas aeruginosa*. It is noteworthy that the silver nanoparticles generated in our study showed no signs of toxicity, unlike those reported in other studies. Additionally, the silver nanoparticles biosynthesized by bacteria in our research were deemed safe and non-toxic.

## Data Availability

The data used to support the findings of this study are included within the article.
